# A Vibration Analysis of the Rubber Inertial Dampers Used in Electrical Vehicles

**DOI:** 10.3390/polym14050953

**Published:** 2022-02-27

**Authors:** Calin Itu, Sorin Vlase, Marin Marin

**Affiliations:** 1 Department of Mechanics, Transilvania University of Brasov, B-dul Eroilor, 29, 500036 Brașov, Romania; calin.itu@unitbv.ro; 2 Romanian Academy of Technical Science, B-dul Dacia 26, 030167 Bucharest, Romania; 3 Department of Mathematics, Transilvania University of Brasov, B-dul Eroilor, 29, 500036 Brașov, Romania; m.marin@unitbv.ro

**Keywords:** suspension, damping, electrical vehicle, rubber

## Abstract

The development of electric vehicle manufacturing, which is considered a useful new popular propulsion system, has major design differences compared to conventional vehicles. This requires a reconsideration of the main components of vehicles and an analysis of them to determine the optimal design and solutions for the new models of cars. Among the many systems that need to be reconsidered is the suspension. A cheaper solution for reducing the car’s vibrations is suspension where the damping is ensured by elastic rubber elements, which are very simple, as they have significant structural damping and a much lower price than the classic solution. The main advantage of this solution is the simplicity. The paper presents and analyzes such an element, analyzing the vibrations of this element and the way in which inertial masses (metal spheres) inserted into the volume of the rubber influence the behavior of this element. The transmissibility of such an element, and how the number of balls and the level of structural damping influences this property, is also analyzed. The results suggest possible applications in the automotive industry.

## 1. Introduction

The solution proposed for the use of inertial dampers ensures, through the correct structural design and dimensioning, the dissipation of the mechanical energy generated by the sources of vibrations or noise. The idea of using such dampers is not new, but the application in the automotive industry is not very common. This type of shock absorbers, as a result of the stability of the higher dissipative power (mainly due to the distributed system that allows high-energy absorption), ensures good performance in operations and a low manufacturing price. At the same time, due to the uniform spatial distribution of the inertial masses, a more uniform thermal load is ensured, thus allowing the appropriate dissipation of the energy introduced into the system. In this way, the demands decrease, and the possibility of the appearance and development of some micro-cracks is reduced. Thus, these devices can become excellent shock absorbers with possible applications in the automotive industry.

The use of rubber to make different types of shock absorbers has been done for a long time, and there is rich literature describing the advantages of using such solutions. Various engineering applications of rubber shock absorbers are currently being studied.

For example, a viscoelastic model was used for the dynamic study of high-speed rail vehicles with equipment suspended under the chassis (UCE) [1]. In this study, it was found that the decrease in the ambient temperature leads to an increase in the dynamic rigidity of the elements used, which ultimately affects their operation. This leads to more intense vibrations, especially for low modes. At high ambient temperatures (around 40 °C), it was found that the dynamic stiffness and the damping coefficient decreased, thus decreasing the damping effect.

Many articles have presented methods for calculating and analyzing the damping systems used in the case of motor vehicles [2,3,4,5]. The studies described the design, modeling, simulation, and the study of the performances of the damper used in the cars. The driver’s seat comfort is an important parameter that was take into account. Different designs of suspension were analyzed and studied in order to respect the requirements of the industry.

Types of shock absorbers, with inertial elements, were used in the seismic attenuation system. The study of such a system was done using the finite element method (FEM). The natural frequencies are significantly influenced by the rigidity and inertial properties of the additional elements introduced into the mass of the damper [6]. The stabilizing properties of a rotary inertial shock absorber have been shown to be excellent in terms of displacement control, as well as acceleration. The influence of the inertia of the additional elements has been proven to have a reduced effect, but their rigidity has a significant effect on the behavior of the structure.

There are many theoretical models to study these systems. The basis for the elaboration of a complex model of a shock absorber, with high density masses incorporated into the elastic material, are presented in [7]. A more elaborate model is presented in [8] where such damping systems are considered as “meta-materials”, or hybrid mechanical (composite) structures. 

An original model that uses the vascoelastic properties of materials to analyze the behavior of rubber in the nonlinear field is presented in [9]. The effect of the operating temperatures, the frequency, and the amplitude of vibrations that occur in the case of high-speed railway vehicles are also considered. It was found that the decrease in the ambient temperature leads to a significant increase in the dynamic rigidity of the elements made of rubber. Of course, such an effect impacts the functionality of these elements. Thus, the vibrations that appear will increase the energy of the low vibration modes, degrading the quality of the operation of the railway vehicle. If the ambient temperature increases, the dynamic stiffness and the damping coefficient decrease, which will lead to a decrease in the damping effect and an increase in amplitudes.

It is known that the laminated high damping elastomeric bearings, used for engine isolate mounts, is a common solution in automotive and aerospace applications. This solution offers a simple means of isolating the structure from the engine vibration. There is a large class of elastomers, such as natural rubber (NR), Neoprene (CR), Poly(Methyl Methacrylate (PMMA), polyurethane (PU), etc [10]. A detailed study of the key properties of the materials as a dynamic modulus, and for damping, is made in the paper and some engineering solutions are proposed.

The damping properties are designed according to the specific needs of the system that the damper is included in. This can become important in the case of electric vehicles. There are two sources of vibrations in electric cars that lead to the existence of two distinct frequency bands. First, there is a range of low frequencies, generated by the electric grinder. A second interval is due to the characteristics of the electrical equipment, with higher frequencies, such as those produced by the electrical switching circuit. For this second area, the inertial damper can give very good results. 

An experimental investigation of the rubber engine mount’s vibration and noise was studied in [11]. Rubber is a common material used for supporting and damping requirements. The work included an experimental analysis of some types of rubber mounts, and the results were compared with that of the existing rubber mount. Therefore, it is possible to improve their performance. Different constructive solutions and the construction of advanced computational models are addressed in [12,13,14]. A detailed study of butyl rubber-based vibration damping formulations is made in [15]. The paper tried to eliminate the ecologically unfriendly aromatic compounds and suggested a new type of compound which has the same properties, but at a lower cost. This new material that was proposed and studied can be used in automotive engineering, and the aircraft industry, for an effective vibration isolation.

Usually, the engine mounting that normally exists in engineering applications are elastomers of which rubber has a good damping property in vibration isolation. In [16,17], experimental investigations were made with the natural rubber mount and other materials in order to optimize the engine mount. The recycling of used car tires and plastic bottles (i.e., polyethylene terephthalate, colloquially called PET) was used to produce the composite materials used dynamic interactions involving vibrations [18]. The paper had an interest in developing composite materials with defined properties used in mechanical engineering, in this case, to reduce the vibration. The noise source of a car and, in particular, of an electric vehicle, is varied. This means that there is a large range of frequencies from, low-frequency to high-frequency distributions. In the paper [19,20], a composite used for the noise reduction with damping and acoustic absorption properties was study for the control of the vehicle’s interior noise. The research showed significant improvements in the noise, vibration, and harshness performance.

Experimental analyses of the damping systems used in automotive engineering are presented in [21,22]. 

The use of electric vehicles requires major changes in the conception and design of such vehicles, and some of the existing components in classic vehicles disappear because they are no longer needed, or their operating parameters are dramatically changed [23,24]. This is the case with the suspension of such a car. In the absence of the internal combustion engine, which is a major and continuous exciter, the electric engine is much quieter and does not have large variations in its movement during operation. As a result, the suspension of such a vehicle, and its components, undergo major changes in design. 

In this paper, we aim to study how suspension with rubber elements can be used to dampen the vibrations generated by the road, in the case of an electric vehicle. The results offer arguments for using this type of damper in the manufacturing of the electrical vehicle. The behavior of such a shock absorber is studied in a static case, and then the vibrations of such a damper are determined.

## 2. Model and Methods

**Figure 1:**
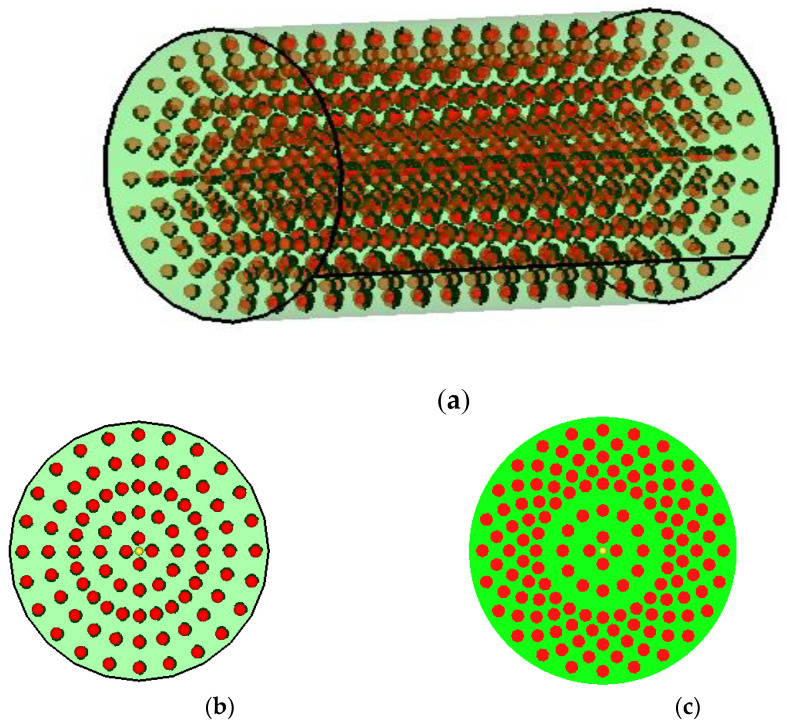
Physical model of the inertial damper. (**a**) Cylinder with balls (**b**) Version I with 1,320 balls. (**c**) Version II with 2040 balls.

**Figure 2:**
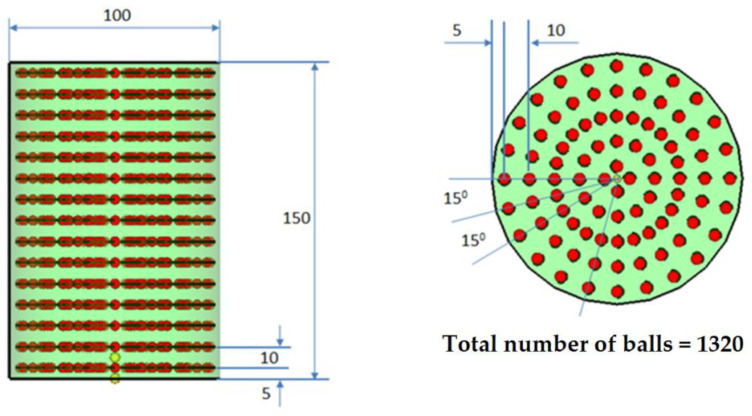
Model no. 1 for the damper with 1320 balls.

**Figure 3:**
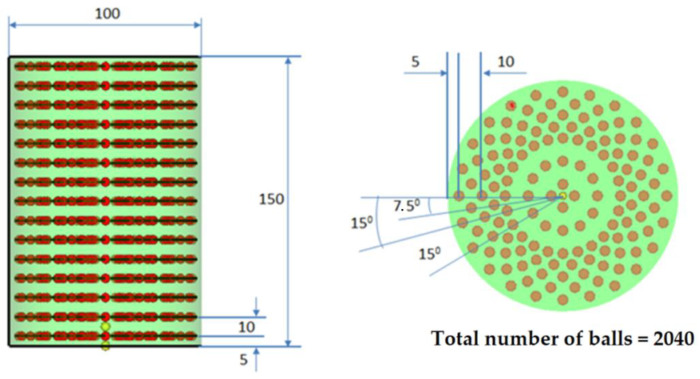
Model no. 2 for the damper with 2040 balls.

The paper will study the dynamic damping and the damping capacity of a shock absorber made of rubber in which inertial elements are inserted. Two principal models of cylindrical dampers are designed and are presented in Figure 1, Figure 2 and Figure 3. In Figure 1, a model of the cylindrical inertial damper used in our research is presented. This element is obtained by reinforcing rubber with steel balls (the number of balls is 1,320). The properties of the two materials used are (values currently used in engineering): rubber, with a Young’s Modulus of 100 MPa, Poisson’s ratio of 0.49, and a density of 2000 Kg/m^3^; and steel, with a Young’ s Modulus of 200,000 MPa, Poisson’ s ratio of 0.3 and a density of 8000 Kg/m^3^.

In Figure 2 (see Table 1) and Figure 3 (see Table 2), the two versions of the model used are presented, including the model with 1320 balls and model with 2040 steel balls.

The properties of the materials used are described as follows. Viscous damping, originally adopted for its mathematical simplicity, must be replaced by a model in which the energy dissipated by damping is independent of the frequency. This type of damping is called structural or hysteretic damping. The term “structural” damping is used in this paper. It involves a force in the opposite direction that is in phase with the speed but, unlike the viscous damping, has an amplitude that is not proportional to the speed but to the displacement. The damping coefficient is inversely proportional to the pulsation, so the damping force is $-h\dot{x}/\omega$ (instead of $-c\dot{x}$), as seen in the following equation:

(1) $$m\ddot{x}+c\dot{x}+kx=F_{o}\cos\omega t,$$

which describes the damped forced vibration of a system with a degree of freedom, which becomes:

(2) $$m\ddot{x}+\frac{h}{\omega }\dot{x}+kx=F_{o}\cos\omega t,$$

where *h* is a structural damping coefficient. The inclusion of the pulsation *ω* in the velocity coefficient $\dot{x}$ implies that the solutions are valid only for this pulsation. The equation of motion (2) can also be written using a complex rigidity (because the condition has a harmonic solution), in the form:

(3) $$m\ddot{x}+\left(k+ih\right)x=F_{o}e^{i\omega t},$$

since *c* is replaced by *h/ω*, the energy dissipated per cycle is:

(4) $$W_{d}=\pi hX^{2},$$

which is independent of pulsation. The amplitude and phase are:

(5) $$X=\frac{F_{o}}{\sqrt{{\left(k-m\omega^{2}\right)}^{2}+h^{2}}}=\frac{X_{st}}{\sqrt{{\left(1-\frac{\omega^{2}}{\omega_{n}^{2}}\right)}^{2}+h^{2}}},$$

(6) $$tg\phi =\frac{h}{k-m\omega^{2}}=\frac{g}{1-{\left(\frac{\omega }{\omega_{n}^{}}\right)}^{2}},$$

where *g* = *h*/*k* is the structural damping factor.

For a system modeled with FEM, with *n* degrees of freedom, the Equation (2) becomes [25]:

(7) $$\left[m\right]\left\{\ddot{x}\right\}+\frac{1}{\omega }\left[d\right]\left\{\dot{x}\right\}+\left[k\right]\left\{x\right\}=\left\{f\right\}e^{i\omega t},$$

Here, [d] represents the structural damping matrix. This matrix is real, symmetric, and positively defined. We have the following relation:

(8) $${\left\{u\right\}}_{s}^{T}\left[d\right]{\left\{u\right\}}_{r}=0\;,\;s\neq r\;\mathrm{and}\;s,r\leq n,$$

With these notations, the modal structural damping coefficients are:

(9) $$D_{r}={\left\{u\right\}}_{r}^{T}\left[d\right]{\left\{u\right\}}_{r}=0\;,\;r=\bar{1,n},$$

and the modal structural damping matrix is:

(10) $$\left[D\right]=diag\left[D_{r}\right]\;,\;r=\bar{1,n},$$

If:

(11) $$K_{r}={\left\{u\right\}}_{r}^{T}\left[k\right]{\left\{u\right\}}_{r}=0\;,\;r=\bar{1,n},$$

the modal structural damping factors are:

(12) $$g_{r}=\frac{D_{r}}{K_{r}}\;,\;r=\bar{1,n}.$$

**Figure 4:**
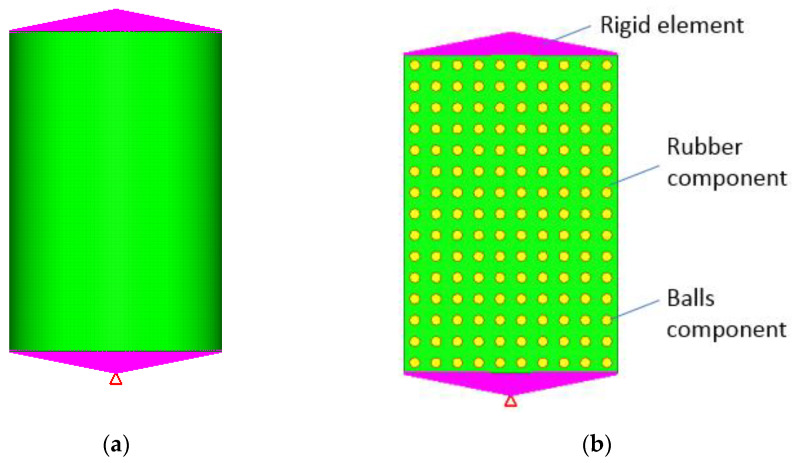
Finite element model of the damper. (**a**) Entire model, (**b**) Section.

To study our models, the FEM is used. The model [26,27,28] consists in two components: the rubber component, and the balls components, which are connected to each other through common nodes techniques (Figure 4). At both ends, two rigid elements are used to apply constraints to the model, and the excitation is considered as a unitary displacement. The total number of nodes and elements on each component are as follows: the rubber component (280,057 nodes and 258,120 elements) and the balls components (35,640 nodes and 10,560 elements). The type of elements used in the simulation are the first-order hexahedral elements with eight corner nodes. 

**Figure 5:**
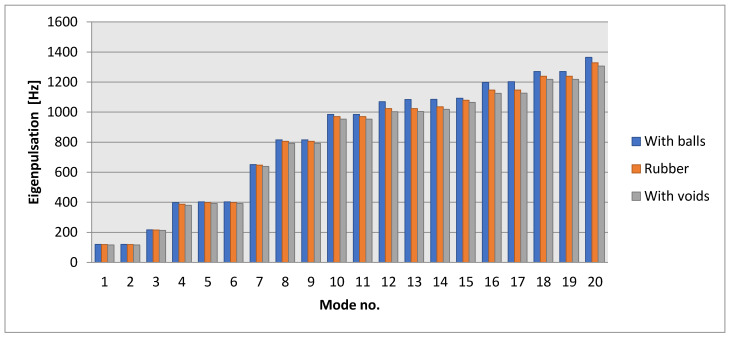
Eigenpulsations for three different cases.

The following is a calculation of the shock damper’s eigenpulsations in the case of a 6% structural damping. This was the case in which 1320 steel balls were placed inside the damper. Three cases were analyzed. The first is where the steel balls were arranged inside the shock absorber; the second is where the eigenpulsations were obtained in case the shock absorber was made only of rubber; and the third case considered a rubber shock absorber with voids (i.e., air spaces), instead of balls. The obtained eigenpulsations, calculated with FEM, are presented in Table 3 and Figure 5.

By analyzing the values obtained in the three situations, it can be observed that the eigenpulsations are quite close, which shows that the use of steel balls in the body of the shock absorber will not greatly influence the spectrum of eigenfrequencies.

A new version in which a number of 2,040 balls were considered inside the same type of shock absorber led to the values presented in Table 4.

In this case, it too can be observed that the use of inertial elements in the rubber mass practically does not influence the calculated eigenpulsations. It turns out that while these masses may have beneficial influences in terms of transmissibility, they do not influence behavior in resonant areas. Therefore, it is not necessary to redesign such a system to take into account these influences.

**Figure 6:**
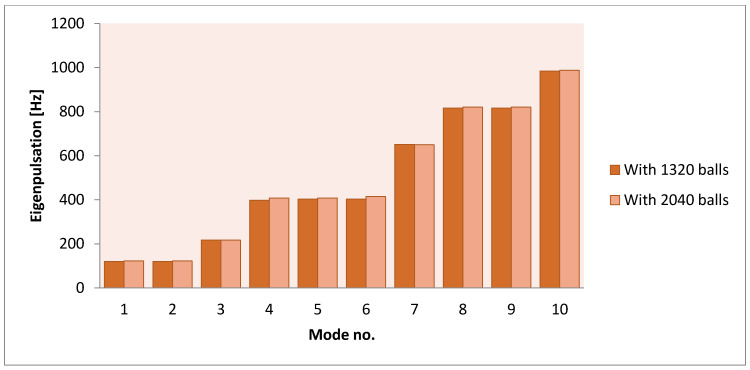
Comparison of the eigenpulsations obtained for the two studied models.

A comparison between the two cases studied, with 1320 steel balls and with 2040 balls shows that, practically, the obtained values are the same (Figure 6).

The observation made in the previous case remains if the number of the balls increase. The eigenpulsations remain, practically, unchanged. It turns out that they do not influence behaviors in resonant areas. 

What is interesting about these types of elements is their damping capacity. We will study this capability by analyzing the transmissibility of such a system. This analysis is made for several variants in the next section.

## 3. Results

To determine the effect of using inertial dampers with steel balls included in the rubber, as well as the effect of the number of balls on the vibrations, the calculation of the transmissibility of the shock absorber is performed, which is defined as the ratio between the amplitude of a sinusoidal oscillation of the cylinder base points and the amplitude of the stationary response of the points on the other base. In the case of this application, a displacement of 1 mm was imposed on one of the bases and the amplitude of the oscillations of the other base in mm was determined. The size of this amplitude, measured in millimeters, is the value of transmissibility (displacement).

**Figure 7:**
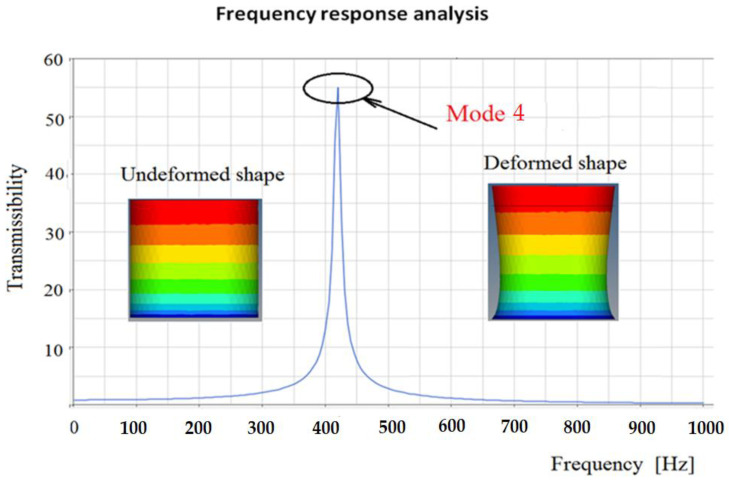
Frequency response for a rubber model, with a structural damping of 2%.

**Figure 8:**
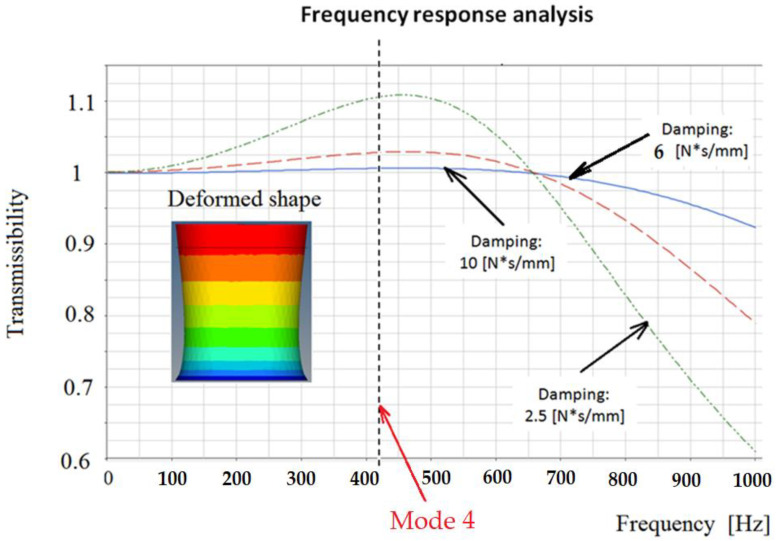
Frequency response for an inertial damper with steel balls.

In Figure 7 the transmissibility calculated for a damper with 1,320 balls with a structural damping of 2% is presented. Here, it is easy to see a resonant behavior around the eigenfrequency 4. If it is considered a viscous damping, the three cases are presented in Figure 8. From this figure, it is obvious that a viscous damping can reduce the transmissibility significantly. However, to obtain this viscous damping, it is necessary to use engineering solutions that are expensive considering the design and manufacture. A solution is to use the structural damping offered by the material itself, instead of using other expensive devices.

**Figure 9:**
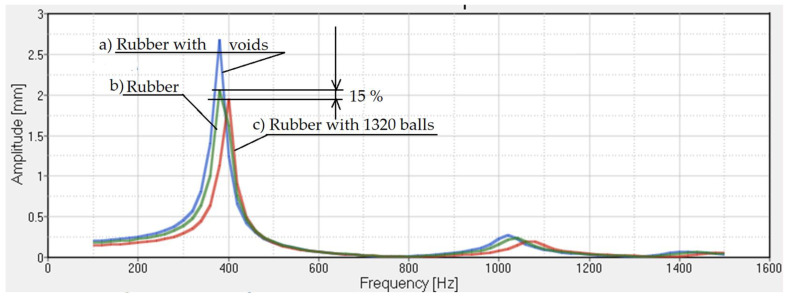
Transmissibility for: (**a**) rubber with voids; (**b**) rubber; and (**c**) rubber with steel balls. The structural damping is 6%.

We will study the transmissibility of the damper if we only use a rubber damper with steel balls. In Figure 9, the transmissibility obtained using a rubber damper with 1320 balls is presented. It is easy to observe that the use of the balls inside the rubber can decrease this size by 15%, while the spectrum of eigenvalues remains virtually the same.

**Figure 10:**
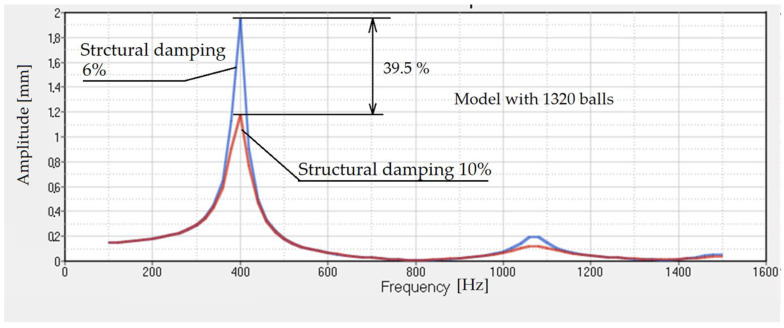
Transmissibility for rubber with 1320 steel balls. The structural damping is 6% and 10%.

The results are obtained using a material with 6% structural damping. If we increase this damping to 10%, the results are presented in Figure 10. The important effect that increasing structural damping has on the transmissibility of the damper is obvious.

**Figure 11:**
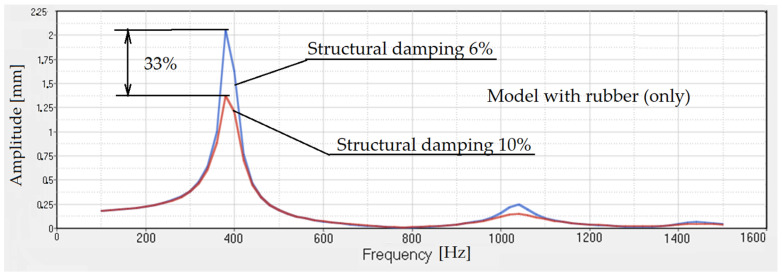
Transmissibility for rubber without balls. The structural damping is 6% and 10%.

**Figure 12:**
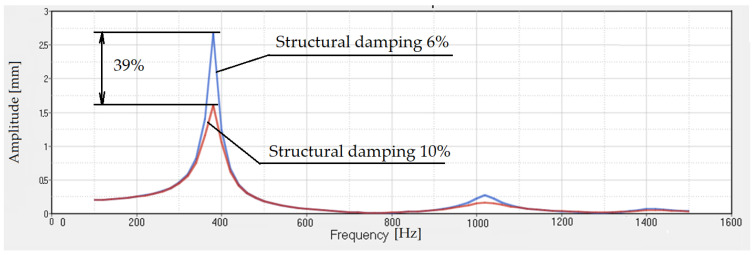
Transmissibility for rubber with voids. The structural damping is 6% and 10%.

If we do the same study for rubber only (Figure 11) or for rubber with voids (Figure 12), the same conclusions can be drawn.

**Figure 13:**
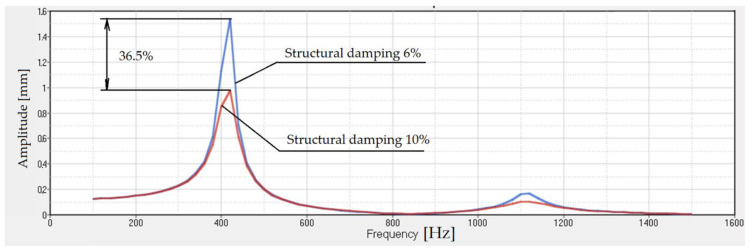
Transmissibility for rubber with 2040 steel balls. The structural damping is 6% and 10%.

The results obtained by increasing the number of the steel balls, in the same volume, are presented in the Figure 13. There, two cases were analyzed, one using a 6% structural damping, and the other considering a 10% structural damping. We observe the same important decrease in the transmissibility as in the case of 1320 balls. It obtained a 39% decrease in transmissibility.

**Figure 14:**
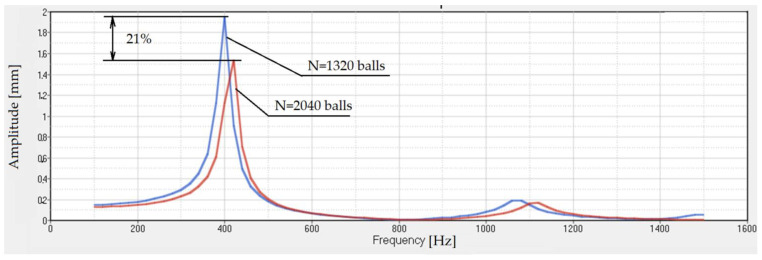
**Comparasion** Transmissibility for structural damping 6%.

A comparison between the two types of dampers, with 1320 and 2040 balls, is shown in Figure 14. It can be seen that the increase in the number of inertial elements leads to a decrease in transmissibility. Inserting metal elements into the rubber body is a cheaper operation than using expensive damping devices.

## 4. Conclusions

Two models of shock absorbers made of rubber were studied in this paper. It has been shown that the inertial elements introduced inside the rubber can positively influence the damping capacity. These dampers can be a convenient solution for vibration damping in the body of an electric car. This occurs without negatively influencing the other vibration properties of the system. Their appearance implies a rethinking and a redesign of the main aggregates and subassemblies of such a car. For this, several variants of rubber shock absorbers were studied in which inertial elements were introduced, namely steel balls. The calculation shows that the existence of balls in the body of the shock absorber can lead to a decrease in transmissibility, which is a positive element for our study. It can also be seen that the increase in the number of inertial elements also leads to a decrease in transmissibility. Therefore, the increase in the number of inertial elements leads to an improvement in the properties of the vibrations of the system. Inserting metal elements into the rubber body is a cheaper operation than using expensive and difficult-to-manufacture damping devices.

It can be concluded that these types of shock absorbers can be considered as a feasible solution, in terms of damping qualities, when designing electrically powered vehicles. They are a simple construction solution, with a low cost, and are easy to maintain.

## Figures and Tables

**Table 1:** The geometry of balls. N = 1320 balls.

No. of Radial Rows	No. of Balls	Angle
1	4	90°
2	12	30°
3	24	15°
4	24	15°
5	24	15^o^

**Table 2:** The geometry of balls. N = 2040 balls.

No. of Radial Rows	No. of Balls	Angle
1	4	90°
2	12	30°
3	24	15°
4	24	15°
5	24	15°
6	24	15°
7	24	15°

**Table 3:** The eigenpulsations for three different cases (N = 1320).

Mode No.	With Steel Balls (N = 1320)	Without Balls (Rubber)	With Voids
	The values are expressed in Hz		
1	119.60	118.38	116.27
2	119.61	118.38	116.28
3	216.99	215.78	212.91
4	398.07	387.56	379.47
5	402.66	398.23	391.69
6	402.70	398.23	391.70
7	650.95	647.27	638.66
8	816.04	806.40	792.39
9	816.13	806.41	792.41
10	984.06	971.10	953.08
11	984.14	971.12	953.11
12	1069.14	1023.75	1002.57
13	1083.99	1023.77	1003.42
14	1084.85	1034.82	1018.36
15	1092.35	1078.58	1064.17
16	1197.32	1146.70	1125.29
17	1202.60	1146.72	1125.81
18	1270.41	1239.27	1217.81
19	1270.55	1239.27	1217.84
20	1364.00	1328.27	1306.41

**Table 4:** The eigenpulsations for three different cases (N = 2040).

Mode No.	With Steel Balls (N = 2040)	Without Balls (Rubber)	With Voids
	The values are expressed in Hz		
1	122.47	118.38	116.27
2	122.55	118.38	116.28
3	216.72	215.78	212.91
4	407.58	387.56	379.47
5	407.79	398.23	391.69
6	414.52	398.23	391.70
7	649.91	647.27	638.66
8	820.46	806.40	792.39
9	820.87	806.41	792.41
10	987.31	971.10	953.08

## Data Availability

Not applicable.
